# Quality, Understandability and Reliability of YouTube Videos on Skin Cancer Screening

**DOI:** 10.1007/s13187-023-02320-w

**Published:** 2023-06-15

**Authors:** Lydia Reinhardt, Theresa Steeb, Anika Mifka, Carola Berking, Friedegund Meier, on behalf of the German Skin Cancer Council

**Affiliations:** 1 Department of Dermatology, University Hospital Carl Gustav Carus, TU Dresden, Germany; 2 Skin Cancer Center at the University Cancer Centre Dresden and National Center for Tumor Diseases, Dresden, Germany; 3 Department of Dermatology, Uniklinikum Erlangen, Friedrich-Alexander University Erlangen-Nürnberg, Erlangen, Germany; 4 Comprehensive Cancer Center Erlangen - European Metropolitan Region of Nürnberg, Erlangen, Germany

**Keywords:** Skin cancer, Skin cancer screening, Internet, Videos, Health promotion, Shared decision-making, Patient information, Reliability, Quality

## Abstract

In 2008, a nationwide skin cancer screening (SCS) program was implemented in Germany. However, participation rates remain low. YouTube videos on SCS might educate eligible persons to undergo SCS. Until now, no scientific evaluation of the quality of videos available for German-speaking persons eligible for SCS has been performed. Here, we identified and evaluated videos on SCS provided on YouTube. YouTube was searched in May 2022 for German terms related to SCS. Two authors evaluated the videos of the first three pages that met the predefined eligibility criteria. The quality of the videos´ information was evaluated using DISCERN and the Global Quality Scale (GQS). The understandability and actionability were assessed with the Patient Education Materials Assessment Tool (PEMAT). The reliability was assessed with the Journal of American Medical Association (JAMA) score. Subgroup differences were identified by the Kruskal–Wallis test. Overall, 38 videos were included in the evaluation. Most videos were provided by health professionals (clinics and practices). The average scores (mean (SD)) for the individual tools were as follows: DISCERN 3.1/5 points (± 0.52), GQS 3.72/5 points (± 0.7), understandability 64,27% (± 13.53%), actionability 58.22% (± 15.18%), JAMA 37.17% (± 18.94%). These results indicate a mediocre to good understandability, a mediocre quality and actionability, and a low reliability. Videos that were assessed as useful were of significantly better quality. An improvement of freely available informational videos on SCS, especially with regard to the reliability criteria, is urgently needed.

## Introduction

Skin cancer is the most frequently diagnosed cancer entity. The incidence of melanoma and non-melanoma skin cancer has steadily increased in recent years [1, 2]. Early detection of suspicious skin lesions is the most important strategy in (secondary) prevention, in addition to reducing exposure to ultraviolet radiation by sun protection measures (primary prevention) [3]. Therefore, the national skin cancer screening (SCS) program was introduced in Germany in 2008. It aims at reducing skin cancer-associated mortality and morbidity. Since its introduction, more than 13 million people have participated in the SCS program and estimated participation rates ranged between 24 and 39% [4–7].

Dermatologists as well as general practitioners substantially contribute to SCS. The costs for SCS are reimbursed by all German statutory health insurance companies every two years for members who are older than 35 years [4]. SCS involves a voluntary, standardized full-body examination. Nevertheless, SCS participation rates remain low [4, 5, 8]. One explanation might be that beneficiaries are unaware of the possibility to undergo SCS or that they are afraid of the examination of their entire skin, including intimate parts of the body. Although medical consultations and printed information remain to be the most important sources of health information, a steadily increasing number of persons is seeking health information on the internet [9–12]. YouTube is an open access video-sharing platform, which is increasingly used to disseminate health-related information. This platform has become an easily accessible source for patients to acquire information related to their diseases [13]. However, the distribution of medical information to such a huge audience offers invaluable opportunities but also challenges as the quality of unfiltered information posted can be of low scientific quality [14]. Information may even be misleading or harmful and the credibility of the providers cannot be verified and quality control of these videos has not been established yet [15–17].

Since no scientific evaluation of the quality of videos available for German-speaking persons eligible for SCS has been performed so far, this study aimed to identify YouTube videos on SCS and to assess the quality, reliability, usability, and understandability. The results of this study may encourage shared decision-making and be beneficial for both patients and health care providers, helping them to recommend appropriate videos to their patients and thus, also increase participation rates in SCS.

## Material and Methods

### Search Strategy

A video search on YouTube was conducted in May 2022, using German SCS-related keywords. The standard search options provided by YouTube were maintained. The first three pages, i.e. 60 videos, were searched by two independent researchers (TS, LR) for each keyword. A significant proportion of users has been shown to watch videos from only the first three pages [18].

### Eligibility Criteria

Videos had to meet the following inclusion criteria to be eligible for evaluation: contain information referring to SCS; be accessible for free and for all users; and be in German language. Videos were excluded if they were commercials, without sound, presented only photos, or if the duration was less than one minute. All search results were screened for duplicates, and the predefined eligibility criteria were applied.

### Grouping of Videos

Due to the variety of the video providers, the videos were grouped following a previous categorization used in the evaluation of videos on skin cancer [14, 19, 20]. Two researchers (LR, AM) categorized the videos according to their original source into the following categories: layperson, health professionals (hospitals, practices), health insurances, education, non-commercial providers/professional society, pharmaceutical company, health portal, and unclassified. For television or news reports, we distinguished whether they were uploaded by the official channel or whether they were re-uploaded by private providers.

### Data Management

The available baseline information (URL, title, name of the provider, length, and year of upload) of each selected video was documented. Additionally, the number of views, likes, and dislikes was extracted. The baseline information was extracted to an internally piloted data extraction sheet using Microsoft Excel 2010. Two reviewers (LR, AM) independently assessed the videos’ quality of information, reliability, and understandability. Prior to the assessment, the use of the assessment tools was piloted by independently evaluating the first five videos to discuss potential difficulties and resolve questions.

### Quality of Information

The DISCERN tool is commonly used to assess the quality of cancer information and was developed for laypersons [21]. A modified German version of this tool was used, consisting of 9 items to review: video’s transparency (items 1–6); content (items 7–8); and to give an intuitive assessment summary (item 9). Items were scored on a 5-point scale ranging from 1 (“criterion is not met at all”) to 5 (“criterion is fully met”). A maximum of 45 points could be achieved.

Additionally, the Global Quality Scale (GQS) was used. The GQS includes a 5-point scale ranging from 1 (low) to 5 (high quality) [22]. For both tools, videos with a mean score of ≥ 4 points were considered to be of good quality, < 4 and ≥ 2 points of medium quality, and < 2 points of low quality.

### Understandability and Actionability

The Patient Education Materials Assessment Tool for Audiovisual Materials (PEMAT-A/V) was chosen to assess the individual videos’ understandability and actionability [23]. The understandability section comprises 13 items that covered content, word choice and style, organization, layout and design, as well as the use of visual aids. The second section covers actionability by 4 items, meaning that users can identify actions, they can take with the information [23]. Each item can be scored as 0 (“disagree”), 1 (“agree”), or N/A (“not applicable”). PEMAT scores range from 0 to 100%, with higher values generally indicating better understandability or actionability.

### Accuracy, Utility, and Reliability

The accuracy, utility, and reliability of each video source were evaluated according to the Journal of American Medical Association (JAMA) benchmark criteria [24]. These four criteria included authorship (authors, contributors, affiliations, credentials), attribution (references and sources used for the content and copyright information), disclosures (sponsorship, advertising, commercial funding, potential conflicts of interests), and currency (dates of posted and updated information). Each item can be scored as 0 (“disagree”) or 1 (“agree”). The higher the score, the more accuracy, utility, and reliability items were fulfilled.

### Harms and Benefit

In order to summarize their potential benefit or harm, the videos were rated on an adapted 3-point scale whether they were perceived to be useful, neutral, or harmful for potential audiences [14, 19, 20]. Videos were judged to be useful if they contained accurate information, whereas videos were judged harmful if the videos contained inaccurate or misleading information.

### Statistical Analysis

Statistical analyses were conducted by using SPSS (IBM SPSS Statistics version 28, IBM Corporation, Armonk, NY, USA). Descriptive analyses included mean (SD) or median and interquartile ranges (IQR). Subgroup differences were explored using the Kruskal–Wallis test. The relationship between the individual items of the tests was examined using Spearman’s correlation. Statistical significance was set at *P* ≤ 0.05. The inter-rater agreement of the two reviewers was determined using the intraclass correlation coefficient, as well as by determining the inter-item correlations r between the individual reviewers.

## Results

### Video Identification and Baseline Characteristics

**Fig. 1:**
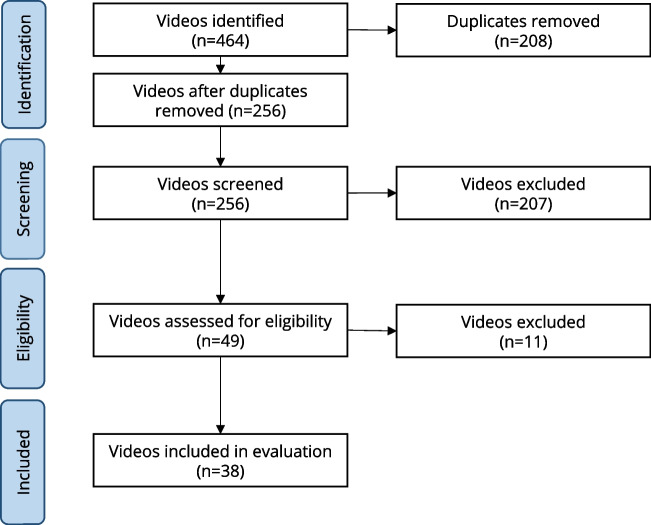
Flowchart showing the video identification process for the 38 YouTube videos on skin cancer screening

Our search identified 464 videos. Two reviewers (TS, LR) screened the videos for duplicates and checked them for compliance with the pre-defined eligibility criteria. Finally, 38 videos were considered for assessment (Fig. 1). Most videos were provided by health professionals (39.5%, 15/38), followed by TV reports uploaded by official TV channels (18.4%, 7/38). Furthermore, 13.2% of videos (5/38) were offered by non-commercial providers/professional society and 10.5% (4/38) were provided by laypersons. The remaining videos were uploaded by health insurances, TV reports uploaded by private channels, unclassified providers (5.3%, 2/38, respectively), and one health portal (2.6%, 1/38).

The videos were uploaded between 2010 and 2022 with the majority (19/38) uploaded after 2020 (Table 1). The videos were clicked between 46 and 562,425 times, with a mean of 27,026 views. The length ranged from 1:25 to 70:15 min. The number of likes ranged from 0 to 654 with a mean of 105. Most likes were given on a video uploaded by a private channel, interviewing a dermatologist about moles and SCS (video #6). No dislikes were given to any of the videos.

**Table 1:** Overview of baseline characteristics, quality, understandability, actionability, accuracy, utility, and reliability of the videos according to the provider's categorization

Characteristic	All videos	Layperson	Professional society/ non-commercial provider	TV	TV reports uploaded by private channels	Health Portal^a^	health professionals (hospitals, practices)	Health insurance company	Unclassified
Number videos (%)	38 (100%)	4 (10.5%)	5 (13.2%)	7 (18.4%)	2 (5.3%)	1 (2.6%)	15 (39.5%)	2 (5.3%)	2 (5.3%)
Mean views (SD)	27,026.34 (93,685.33)	15,043 (23,207.76)	135,669.6 (243,725.57)	23,638.43 (43,510.24)	3973.5 (4958.94)	2019	2473 (3830.54)	34,351 (48,018.21)	3624.5 (4735.45)
Mean video length, min (SD)	9.13 (12.53)	10.1 (6.66)	16.96 (30.53)	11.06 (9.42)	19.77 (19.01)	5 .9 (0)	5.2 (3.46)	3.16 (1.35)	7.26 (0.18)
Year of upload (range)	2010–2022	2019–2022	2010–2021	2012–2021	2012–2016	2020	2015–2022	2015–2021	2010–2019
Mean likes (SD)	105.16 (192.73)	335.5 (364.43)	191 (265.29)	169.57 (195.16)	7 (5.66)	15 (0)	24.53 (46.97)	55 (76.37)	2.5 (2.12)
Mean dislikes (SD)	0	0	0	0	0	0	0	0	0
Mean DISCERN points: quality (SD)	3.1 (0.52)	2.66 (0.6)	3.66 (0.49)	3.15 (0.38)	2.94 (0.39)	3.33 (0)	3.05 (0.47)	2.89 (0.94)	3.14 (0.51)
Mean GQS points: quality (SD)	3.72 (0.70)	3.13 (0.63)	4.1 (.65)	4.07 (0.73)	3.5 (0.71)	4 (0)	3.6 (0.63)	3.5 (1.41)	4 (0.71)
Mean PEMAT: understandability (SD)	64.27% (13.53)	59.62% (18.45)	70.7692% (18.57)	66.48% (8.22)	63.46% (8.16)	50% (0)	65.13% (13.2)	51.92% (2.72)	63.46% (24.48)
Mean PEMAT: actionability (SD)	58.22% (15.18)	62.5% (17.68)	60% (27.1)	60.71% (13.36)	50% (0)	70% (0)	57.5% (13.2)	43.75% (8.84)	56.25% (8.84)
Mean JAMA: Accuracy, utility, reliability (SD)	37.17% (18.94)	37.5% (22.82)	60% (27.1)	35.71% (13.36)	25% (0)	0%	35% (10.77)	50% (0)	18.75% (26.52)

*Abbreviations*: *GQS* global quality scale, *PEMAT* Patient Education Materials Assessment Tool, *JAMA* Journal of American Medical Association (JAMA) score, *SD* standard deviation

^a^Only one video was evaluated in this category

Videos that met the inclusion criteria were included regardless of their number of views, which may be influenced by channel popularity, upload date, and search algorithm. If a video additionally contained other information than on SCS, only the corresponding part on SCS was evaluated. Therefore, long videos were also included in the search.

### Quality: DISCERN and GQS Results

Out of 45 points in total, the 38 individual videos ranged between 18.2 and 40 points according to DISCERN. The mean DISCERN scores ranged from 2.02 to 4.44 points with a mean score of 3.1 (± 0.52), indicating a mediocre quality (Table 1). The mean GQS score was 3.72 points (± 0.7), which also indicates a mediocre quality.

### Understandability and Actionability: PEMAT Results

The average PEMAT score were 64.27% (± 13.53%, range 38.46–96.15%) for understandability and 58.22% (± 15.18%, range: 25–100%) for actionability (Table 1). Most score deductions for the understandability domain were due to lack of a summary and lack of visual aids. For the actionability domain, information was often missing on how to break down each action into manageable explicit steps.

### Accuracy, Utility, and Reliability: JAMA Results

In total, 37.17% (± 18.94%, range: 0–100%) of the JAMA benchmark criteria were fulfilled, indicating rather poor reliability (Table 1). The main reasons for score deductions were missing information on the currency of videos, i.e., the upload date, and missing disclosure of the provider. For these items, only one video received full points from both raters.

### Harms and Benefit

The majority of videos (76.3%, 29/38) was evaluated to be neither beneficial nor harmful. 18.4% (7/38) of the videos were evaluated to be useful. The remaining videos were rated as harmful (5.3%, 2/38), but this rating was only given by one of the two raters in each case. The two videos that received the worst ratings were uploaded by a private practice and a layperson. The videos contained misleading information on skin cancer prevention and the funding of SCS.

### Inter-rater Agreement

We determined intraclass correlation coefficients of 0.954 to 0.990 with a Cronbach’s alpha of 0.978, indicating a high overall inter-rater agreement concerning the assessment by DISCERN, GQS, JAMA, and PEMAT. The inter-item correlation r was 0.964, attesting a high individual agreement among the two reviewers when assessing the individual items.

### Subgroup Analysis

Videos rated as useful showed a significantly better quality in comparison to those rated as neutral (DISCERN: *p* = 0,001; GQS: *p* = 0,002) or harmful (DISCERN, GQS: *p* = 0,001). No further subgroup differences were identified.

### Correlation Analysis

Significant positive correlations were identified between the formal quality ratings and the rating of the videos as useful, neutral or harmful (DISCERN *r* = 0,702, *p* < 0,001; GQS *r* = 0,687, *p* < 0,001). A strong correlation was found between the two quality tools DISCERN and GQS (*r* = 0.838, *p* =  < 0,001). A mediocre correlation was also found between the assessment of the videos´ usefulness and understandability (*r* = 0,418, *p* = 0,009).

## Discussion

In this study, 38 freely available German YouTube videos on SCS have been systematically identified and evaluated by two independent reviewers. The evaluation was based on tools for quality, usability, actionability, and reliability. The search resembles the process that an interested layperson or patient might perform [18]. The videos were clicked more than 56,000 times at the time of the search, which suggests at least some reach of the topic on one of the most popular online platforms for videos.

**Fig. 2:**
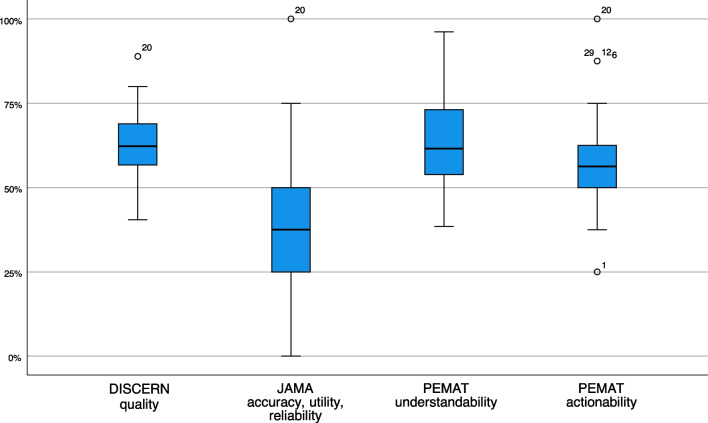
Boxplots showing the quality, accuracy, utility, reliability, understandability, and actionability of 38 videos providing information on skin cancer screening. Box, lower line: quartile Q1 (25% quantile), middle line: median (50%), upper line: Q3 (75% quantile). JAMA = Journal of American Medical Association (JAMA) score; PEMAT = Patient Education Materials Assessment Tool

In general, videos are a suitable source of health information as they combine language and images. Compared to written texts such as brochures, they offer the possibility to present facts in a more vivid way and thus also reach people who feel deterred by long texts or who cannot read. However, we found that the videos had some shortcomings with respect to the criteria examined. The worst ratings were given for deficiency of information on currency, sources and references. Strikingly, almost no video met these criteria completely. Content quality was rated mediocre overall, with no video receiving all possible points here either. Actionability was also rated mediocre. Compared to the other criteria, the videos' usability was rated best, particularly for using everyday language (Fig. 2).

This evaluation of YouTube videos on SCS thus complements previous evaluations of skin cancer videos [14, 19, 20]. The comparison shows that reliability was also rated worst here. Since laypersons are not necessarily able to assess whether medical information is correct, it is all the more important to disclose sources, authors and the currency of the data. Although an attractively produced video can arouse interest, it must meet appropriate quality criteria as health information. The videos examined here met these criteria only in part.

Compared to the previous video reviews on different skin cancer entities, no video uploaded by a pharmaceutical company was found in this search, but videos from health insurance companies were found. Pharmaceutical companies might have less interest in videos on SCS, as they cannot market products here. In contrast, health insurance companies have an interest in prevention, which may be a reason for promoting SCS via informational videos on YouTube.

The personal conversation with the physician is still the most important source of information for cancer patients. Nevertheless, additional information that can be obtained outside the doctor's consultation is of enormous importance [9, 12, 25]. Evidence-based information must be freely available, especially for preventive services when there is no contact to a physician yet. Information materials that clearly present the screening procedure and evaluate benefits and risks can thereby contribute to an informed decision. Freely accessible information material is of great importance, as SCS is still insufficiently used.

However, when creating and/or placing videos, all information about the creator, originator, date of update and sources should be accessible, for example in the info box or in the video credits.

It should be considered whether it might be favorable to make videos available on platforms that are specifically aligned to health topics. One example would be the website "Infoportal-Hautkrebs" (https://www.infoportal-hautkrebs.de/), a platform for skin cancer topics. The information provided there has been evaluated for quality and usefulness by physicians, scientists and patient representatives, while the methodology employed is made transparent. This also provides physicians with a site they can recommend to their patients.

## Strength and Limitations

It should be acknowledged that the videos evaluated had different formats. Videos over an hour in length can of course accommodate more information than explanatory short ones. In addition, legal regulations, such as the coverage of costs for the dermatoscope, have changed in recent years. As a result, videos that still contained correct information at the time they were created may now be outdated. Therefore, the comparability between the videos is limited.

## Conclusion

Overall, the videos identified here on SCS revealed a mediocre to good understandability, a mediocre quality and actionability, and a low reliability. Videos that were assessed as useful were of significantly better quality. An improvement of freely available informational videos on SCS, especially with regard to the reliability criteria, is urgently needed.

## Data Availability

Data available on request from the authors: the data that support the findings of this study are available from the corresponding author (LR), upon reasonable request.
